# Factors Associated with Quality of Life in Patients with Type 2 Diabetes of South Benin: A Cross-Sectional Study

**DOI:** 10.3390/ijerph19042360

**Published:** 2022-02-18

**Authors:** Halimatou Alaofè, Waliou Amoussa Hounkpatin, Francois Djrolo, John Ehiri, Cecilia Rosales

**Affiliations:** 1 Department of Health Promotion Sciences, Mel and Enid Zuckerman College of Public Health, The University of Arizona, Tucson, AZ 85724, USA; jehiri@email.arizona.edu; 2 School of Nutrition and Food Science and Technology, Faculty of Agricultural Sciences of the University of Abomey-Calavi (FSA-UAC) Campus d’ Abomey-Calavi, Calavi 01 BP 526, Benin; amouswal@yahoo.fr; 3 Faculty of Health Sciences of the University of Abomey-Calavi (FSS-UAC), Calavi 01 BP 526, Benin; fdjrolofss@yahoo.fr; 4 Division of Public Health Practice & Translational Research, 550 E. Van Buren Street, University of Arizona, Phoenix Plaza Building, Phoenix, AZ 85006, USA; crosales@email.arizona.edu

**Keywords:** type 2 diabetes, quality of life, self-care behaviors, Benin

## Abstract

**Background**: Type 2 diabetes (T2D) adversely affects health-related quality of life (QoL). However, little is known about the QoL of diabetic patients in Benin, where the disease is a growing concern. Thus, this study aims to assess the QoL and its associated factors among T2D patients in Cotonou, southern Benin. **Methods**: A total of 300 T2D patients (age > 18 years) were enrolled, and the diabetes-specific quality of life (DQoL) and Natividad self-care behaviors’ (SCB) instruments were used for data collection. DQoL scores were calculated, and factors associated with DQoL explored using logistic regression. **Results**: The mean of patients’ DQoL was 38.1 ± 4.1, with 43% having low QoL. In terms of DQoL, 56.3% reported a high diabetes impact, followed by low life satisfaction (53%) and high worry about diabetes (32.7%). In the logistic regression analysis, education, marital status, occupation, family history of diabetes, complications, and social support were associated with DQoL. SCB factors, including healthy eating, problem-solving, coping strategies, and risk reduction, were significant predictors of DQoL. **Conclusions**: Patients’ empowerment, starting with self-management education, is essential to improve the QoL of T2D patients in Cotonou. However, the programs need to target low education, low socioeconomic status, low social support, and overweight patients.

## 1. Introduction

Although type 2 diabetes (T2D) is now a worldwide epidemic, the rate of increase in its prevalence in low- and middle-income countries (LMICs) such as those in Sub-Saharan Africa (SSA) is alarming [1,2]. By 2045, the number of adults with T2D is expected to increase by 75–80% in LMICs, double the projected global increase of 48%, and the most significant increase in SSA (162.5%) [3]. Benin (an SSA country) is no exception as the diabetes prevalence more than doubled between 2008 (4.6%) and 2015 (12.4%), reaching 21.6% in some areas of the country [4]. Between 2007 and 2017, diabetes-related disabilities increased by 55.8%, and diabetes-standardized death rates were 618 for women and 430 for men [5,6]. Further, the burden of T2D in Benin is more likely to increase with the nutritional transition and accelerating urbanization [7]. Thus, T2D imposes a tremendous burden on affected individuals, their families, and healthcare systems in the country, suggesting an urgent need to identify possible interventions to provide optimum healthcare to persons living with diabetes.

Health-related quality of life (QoL) indicators are solid predictors of an individual’s competence to maintain long-term health, well-being, and productivity [8]. Several studies have found T2D patients to have a lower QoL than healthy people due to the high demands of treatment, especially if they develop complications, which increase hospitalization, mortality, and disease burden [9,10,11]. For this reason, improved QoL is regarded as a key goal of all healthcare interventions, including those for T2D management [12]. Furthermore, numerous studies have evaluated the QoL of people living with diabetes across numerous regions, including the United States [13], Europe [14], Asia [15,16,17], and Africa [18,19]. They have identified several associated factors, such as sex, age, financial status, educational attainment, occupations, body mass index (BMI), concomitant risk factors (dyslipidemia and hypertension), treatment therapy, and lifestyle [13,14,15,16,17,18,19]. Self-care behaviors (SCBs) are another factor that affects patient QoL [20,21], and Jeihooni et al. suggested that a better QoL could be achieved by providing health education tailored to the needs of SCBs in patients with diabetes [22].

In Benin, T2D is associated with end-stage kidney disease, erectile dysfunction, diabetic foot, stroke, limb amputation, and renal dialysis [23,24,25]. These symptoms could reduce physical and social activities and, as a result, decrease QoL [21]. A recent study on associated risk factors of diabetes also urged improving the health and QoL of diabetes patients to reduce the social and personal costs for diabetes care in the country [26]. However, no study reported QoL and associated factors among patients with diabetes in Benin during the past decade. Importantly, although previous research has focused on QoL, the level of QoL and its predictors vary due to cultural differences and the use of different measurement tools. Thus, it is critical to identify relevant factors and modifiable predictors of QoL in Benin, so that appropriate and context-specific interventions can be designed to manage diabetes better and improve the QoL in these patients.

Finally, self-care behaviors (SCB) of diabetes are an essential part of controlling the disease and improving their QoL [27]. However, only one study in Iran investigated SCB associated with QoL using four dimensions such as nutrition, physical activity, medications for diabetes, and self-monitoring of blood glucose [27]. In Benin, two studies were conducted on the self-care status of patients with diabetes [28,29]. Difficulties were encountered in compliance with diet (20%), physical activity (55.7%), and glycemic control (7.8%) [28], while only 9.1% of patients had good therapeutic adherence [29]. However, little is known about healthy coping or stress management, suggesting an urgent need to identify SCBs that could affect T2D patients’ QoL. Therefore, this study assessed the QoL and sociodemographic, clinical, and SCB associated factors among T2D patients in Cotonou, southern Benin, to fill this knowledge gap. The diabetes SCB was assessed using the Natividad diabetes self-care behaviors questionnaire, which measures the American Association of Diabetes Educators (AADE) seven SCB essential for successful and effective diabetes self-management [30]. We also used the Diabetes-specific Quality of Life (DQoL) instrument to measure QoL, which converts health states into a single index value that can be compared among countries and used for economic analysis [31,32].

## 2. Materials and Methods

This cross-sectional study is part of formative research to assess the contextual factors of the intervention setting that may influence adherence to recommendations of a Diabetes Self-Management Education in Benin. The data collection was conducted from June to August 2019 in four secondary care centers of Cotonou: two public and two private clinics. The study procedures and response rates are explained elsewhere [33]. In brief, 300 outpatients with T2D completed the three-month assessment. Inclusion criteria were: (1) aged 18 years and above; (2) have been living with T2D for a year or more; (3) willing to give informed consent to participate in the study. However, pregnant patients and those with extreme disease condition that restricts them from responding to questionnaires were excluded. The study was reviewed by the National Ethics Committee for Health Research of Benin and the institutional review boards charged with the Human Subjects Protection Program of the University of Arizona. All participants provided written consent after having the study described to them before data collection activities. This paper reports the QoL and its associated factors among T2D patients in Cotonou, southern Benin, where diabetes prevalence increased from 4.4 to 19% between 2008 and 2015 [4].

### 2.1. Measures

Interviewer-administered questionnaires were used to collect data on all study variables. Sociodemographic and clinical factors were collected on age, sex, religion, level of education, marital status, occupation, duration of diabetes, family history of diabetes, comorbidities, body mass index (BMI), social support, use of healthcare services, diabetes education group class, and having an insurance plan. The diagnosis of comorbidities such as nephropathy, retinopathy, and neuropathy was made through the questionnaire and confirmed on their diabetic registration charts or clinical records.

*Diabetes-specific quality of life (DQoL)questionnaire*: A revised version of the DQoL questionnaire was used to measure participants’ life quality [31]. The questionnaire is one of the most widely used survey tools for assessing the diabetic-specific quality of life. It has been translated and validated into Spanish, Chinese, Taiwanese, Iranian, and Malaysian [31,32]. It has three primary scales (life satisfaction, diabetes impact, and worries about diabetes) with 13 core items. The satisfaction domain was assessed with six questions: time takes to manage diabetes, time spend getting checkups, the time it takes to determine the sugar level, current treatment, knowledge about diabetes, and life in general. Four questions were used to assess the impact domain, including feeling pain associated with the treatment, feeling physically ill, interfering with family life, and limiting social relationships and friendships. The rest of the three questions were to investigate the worry domain: pass out, the body looks different, and get complications. Responses to questions were made with a 5-point Likert scale. Satisfaction is rated from 1 (very satisfied) to 5 (very dissatisfied). Impact and worry scales are rated from 1 (no impact and never worried) to 5 (always impacted and always worried). DQoL profile was categorized as low when the mean score was higher than the population means.

*Diabetes self-care behaviors (SCB)questionnaire*: The diabetes SCB was assessed using the Natividad diabetes self-care behaviors questionnaire (Table S1), which measures the AADE 7 SCB essential for successful and effective diabetes self-management [30]. The initial questionnaire had 62-items: 15 items for the domain of healthy eating, 4 for being active, 10 for monitoring, 6 for taking medication, 4 for problem-solving, 13 for healthy coping, and 10 for reducing risks. Since it is a new study, two endocrinologists and four healthcare physicians validated a selection of items. They revised all of the questions to make sure they are understandable to the patients and culturally adapted to the Benin context. In addition, an exploratory principal component factor analysis (EFA) and confirmatory factor analysis (CFA) were conducted to confirm the ability of the revised version of the diabetes SCB questionnaire in measuring the construct intended and the content validity. The data were split into two samples using a random uniform distribution, one used as the EFA training and the other used for CFA.

EFA’s approach was as follows: (1) EFA was first conducted on individual domains to ensure all of the items were stable in their respective domain. If the number of domains produced was more than one, only the domain with the largest number of items was chosen. (2) Subsequently, EFA was tested for all of the items, which fell under their respective domains (Table S2). A moderate correlation was expected between the domains, and hence, the extraction method “principal axis factors” and rotation method “Promax” were chosen. Factor loadings for EFA analyses are presented in the Table S3.

After reducing the number of questions (*n* = 26), CFA was performed to determine whether the chosen items conformed to the theoretical model. The model fit was further evaluated according to the recommendations by Hu and Bentler [34]: standardized root mean square residual (SRMR) ≤ 0.08, Tucker–Lewis index (TLI) ≥ 0.95, comparative fit index (CFI) ≥ 0.95, and root mean square error of approximation (RMSEA) < 0.05. If the model did not fit the data, necessary modifications were carried out by removing items with low factor loading, high-standardized residuals, and high modification index. Modifications were carried out until the model was reasonably fit as well as theoretically sound (Table S4). Scores were assigned to each item, with a value of 1 reflecting good behavior and 0 a bad behavior. Table S5 summarizes the aggregation rules used to code the good behavior response of each question in reference to the AADE7 SCB framework [35]. Overall, the SCB domains and their respective items were conceptualized as follows:

- Healthy eating domain was examined using two questions: one concerning the frequency of eating away from home—how often they eat their meal away from home (never; one to three times a week; >three times a week), and one question relating grain and starches (potatoes, beans, rice, pasta) portion size—how they will describe their portions of grains and starches (<one cup; one cup; >one cup).
- Being active domain was measured using three questions: whether they exercise regularly (no; yes), frequency of 30 min of exercise (<three times a week; ≥three times a week), and how important is it to be active (0 is not important at all and 10 is very important).
- Two questions characterized the Monitoring domain: a test of blood for sugar and a test of urine for sugar or ketones in the last seven days (no; yes).
- Taking medication domain was examined using three questions relating to their regular consumption of medications for their diabetes as prescribed by their doctors (no, yes), how important is it to take their medications (0 is not important at all and 10 is very important), how sure are they to take their medicines (0 is not sure at all and 10 is very sure).
- Problems solving domain was measured when respondents were asked how they treat a low blood sugar reaction (eat or drink 15 to 20 g of fast-acting carbs; others) and a high blood sugar reaction (get physical; take your medication as directed; follow your diabetes-eating plan; check your blood sugar; do nothing; other).
- Healthy coping domain was examined using two questions relating to how important they were able to problem-solve when being faced with every day and/or challenging decisions (0 is not important at all and 10 is very important), and how they feel they can problem-solve when faced with every day and/or challenging decisions (0 is not sure at all and 10 very sure).
- Risks reduction domain was measured with three questions: whether they have had their blood pressure checked, their cholesterol and triglycerides checked, and hemoglobin A1c test carried out in the last three months (no; yes).

### 2.2. Statistical Analysis

Data analysis was conducted in three main steps using STATA version 14.1 (Stata Corporation, College Station, Texas). First, the sociodemographic and clinical characteristics of the participants were summarized using descriptive statistics. Percentages and frequencies were used for the categorical variables, while mean and standard deviation (SD) were calculated for the continuous variables. Secondly, the association of sociodemographic and clinical factors with DQoL was assessed using sequential regression analyses. Stepwise logistic regression was first performed, and correlations that meet the significance (*p* < 0.2) were retained in the pre-specified model. Third, the association of SCB with DQoL was assessed using multiple logistic regression with forwarding selection (likelihood ratio) of predictor variables. The multiple logistic regression was controlled with sociodemographic and clinical factors found significant in the second step.

All models were estimated in two specifications. In the first specification, the aggregate DQoL was decomposed into the three domains to investigate how they were associated with different factors. In the second specification, the total DQoL score was included to assess factors associated with the overall DQoL. The outcome variable in each specification was binary. The overall DQoL was indicated as ‘low quality of life’ (DQoL score> population mean) or ‘good quality of life (total DQoL score < population means). As for domains, we have low life satisfaction (satisfaction score> population mean), high diabetes impact (impact score> population mean), and high diabetes worry (worry score> population mean). Variables were examined for independence of observations, multicollinearity, linear relationship, and no significant outliers. Odds ratio (OR) with 95% CI and the *p*-value < 0.05 were considered to declare significantly associated factors.

## 3. Results

From the total 300 patients, approximately 91% of them were over 40 years old (mean 54.9 years; SD = 11.3). Most respondents were female (70.7%), Christian (79.3%), married (59.3%), and had a family history of diabetes (61%). The majority of participants had primary education (31.3%) and secondary education (32.3%). Half of them were self-employed (52.3%) and have lived with diabetes for 1–5 years (49.7%). About 49% were overweight/obese, while 34.7%, 46.7%, and 51.7% had retinopathy, neuropathy, and hypertension. Two-thirds reported receiving support from friends and family (66.3%), but only 24% attended the education group class. Finally, one-third of them reported using healthcare services (34.3%) three times a year, while 10% had insurance coverage.

**Participants’ diabetes-specific quality of life:**Table 1 shows the descriptive statistics for the DQoL domains and items in each domain. The mean of patients’ overall DQoL was 38.1 ± 4.1 (out of 65 total points), with 43% of participants with low DQoL. Among the three domains of DQoL, 56.3% of respondents scored higher than the mean in the impact domain, followed by low life satisfaction (53%) and worry (32.7%) domains. Specifically, most participants feel frequently and always pain associated with the treatment (65.7%) and physically ill (64.6%). However, less than 15% reported interference with the family and limited social relationships and friendships. In the satisfaction domain, the majority of patients were dissatisfied with the time it takes to manage diabetes (61%), current treatment (63.3%), and life in general (65.4%). However, only 12% of them were frequently and always worried about passing out, or the body looks different.

**Participants’ diabetes self-care behaviors:**Table 2 shows the descriptive statistics for the 18 items of diabetes SCB. By domain, taking medication was the domain in which most of the participants had good behavior (67%), followed by problem-solving (53.7%) and coping strategies (51%). However, 56% of patients reported taking medication for their diabetes; 54.7% felt they could problem-solve when faced with everyday and/or challenging decisions, while only 24.2% knew how to treat a low blood sugar reaction. Furthermore, healthy eating (40.7%), being active (46.3%), and monitoring (49.7%) were the domains with the lowest number of patients with good behavior. Overall, 53.3% of the patients had good SCB.

**Socio-demographic factors associated with participants’ DQoL:** In the multivariate logistic analysis in Table 3, educated and married patients and those with a family history of diabetes and social support were less likely to have a low DQoL as compared to not educated and unmarried patients and those with no social support (OR = 0.36, 95% CI = 0.19–0.89; OR = 0.52, 95% CI = 0.25–0.83; OR = 0.57, 95% CI = 0.22–0.88, OR = 0.50, 95% CI = 0.25–0.84, respectively). Similar results were observed for life satisfaction (OR = 0.56; OR = 0.39; OR = 0.90, OR = 0.49), diabetes impact (OR = 0.26; OR = 0.41; OR = 0.48, OR = 0.51), and diabetes worry (OR = 0.72; OR = 0.32; OR = 0.61, OR = 0.63), respectively.

However, patients with disease duration >5 years and those with comorbidities were more likely to have low life satisfaction (OR = 2.6; OR = 2.02, respectively), a high diabetes impact (OR = 1.30; OR = 2.26, respectively), high diabetes worry (OR = 1.02; OR = 4.36, respectively), and low DQoL (OR = 1.15, 95% CI = 1.07–2.37; OR = 1.93, 95% CI = 1.22–3.50, respectively). Furthermore, female patients were more likely to have high diabetes impact (OR = 2.41), while overweight/obese patients were more likely to have high diabetes impact (OR = 1.20) and low QOL (OR = 1.34). Finally, Government/non-government employee patients were more likely to be worried (OR = 3.87), while self-employed patients were less likely to have a low DQoL (OR = 0.54, P = 0.04).

**Self-care behavior factors associated with participants’ DQoL:** As shown in Table 4, patients who had good healthy eating and risks reduction were less likely to be dissatisfied (OR = 0.79; OR = 0.67, respectively), have bad diabetes impact (OR = 0.55; OR = 0.34, respectively) and very worried (OR = 0.60; OR = 0.38, respectively) or poor DQoL (OR = 0.45, 95% CI = 0.18–0.88; OR = 0.38, 95% CI = 0.26–0.67, respectively). Similarly, patients with good problem solving and coping strategies had lower odds of having bad diabetes impact (OR = 0.32; OR = 0.53), worries (OR = 0.68; OR = 0.33), and poor DQoL (OR = 0.45, 95% CI = 0.27–0.87; OR = 0.51, 95% CI = 0.34–0.94), respectively.

## 4. Discussion

The burden of T2D in Benin and Cotonou, in particular, is steadily increasing and QoL is one of the critical outcomes used to evaluate the effect of managing chronic diseases such as T2D on health, and it reflects a patient’s physical and psychosocial disease burden [11]. The present study used a revised version of the DQoL questionnaire for the first time in Benin and showed a moderate QoL, while 43% of T2D patients had a low DQoL. Similar studies using DQoL in Iran and India reported a mean total score of 54.6 and 121.78, respectively [9,36]. Another two studies conducted in Ethiopia and Saudi Arabia used different measurement scales and affirmed our findings [9,37]. However, these results should be interpreted with caution when comparing the scores as the QoL value sets for each country depending on the choice of instruments, the number of levels, the quality of diabetes care, or the availability of access to support services. In this context, one of the challenging issues in Benin is that 49% of diabetic patients usually are not aware of their illness until the onset of the complications [15].

Even though our study identified an overall moderate DQoL, more than half of participants reported problems in the impact and satisfaction domains, whereas one-thirds in the worry domain. Specifically, most patients felt the pain associated with the treatment and physically ill. Studies in Mexico and Ghana reported similar results, showing that the physical domain of health-related quality of life of T2D patients was mainly affected [38,39]. This consistency could be justified by diabetes manifesting more physical than psychological, environmental, and social relationship domains. Indeed, in the present study, less than 15% reported interference with the family and limited social relationships and friendships, similar to studies conducted in Malaysia, Ghana, and Iran [38,40,41]. This result could be due to the social support they received (66%). In contrast, a study in Poland found the lowest social domain score, which could be due to sociocultural variations and lifestyle differences [42]. Finally, the worry domain result proves that patients in this study are not affected by their appearance despite being subject to similar forms of stigma as those living with HIV/AIDS in SSA [43].

Similar to our study, several studies found a positive association between domains and overall DQoL and education, marital status, family history of diabetes, and social support [9,44,45,46]. Educated patients could be equipped with better life skills and higher disease management ability. Married people can have high QoL due to their spouse’s supporting role. A previous family history of diabetes is likely to increase awareness of the disease among other family members, while the care and assistance from relatives and friends would enhance patients’ perception of QoL. In the present study, DQoL was negatively associated with disease duration and complications, as observed in Iran [47] and Ethiopia [37], confirming complications could affect patients’ physical and psychological functioning. Diabetes has also more impact on women than men, emphasizing the need for sex-specific approaches in diabetes management [48]. Furthermore, the occupation was associated with DQoL suggesting an improvement in socioeconomic status can improve QoL [49]. Finally, overweight/obesity affected the patients’ DQoL confirming Jing et al. findings that exercise could improve diabetic patients’ QoL [49].

Due to the urgent need to identify SCB that could affect T2D patients’ QoL, we used the Natividad diabetes SCB questionnaire that measures the AADE seven SCB essential for successful and effective diabetes self-management. We employed a statistically driven four-step process to identify the core set of strongly linked items to participants’ SCB in Cotonou. The result was an 18-item shortened scale instrument that covers a range of issues, such as dietary habits (eating out of home and grain and starch portion), problem-solving (treatment of hypoglycemia), and coping strategies (dealing with every day and/or challenging decisions). Past research found that some issues go unexplored in-office visits due to limited time for interaction, provider communication style, and patient discomfort raising issues with the provider [50,51] Thus, this reliable and valid shortened and treatment-focused SCB questionnaire could be effectively integrated into clinicians’ office settings. However, additional research will need to examine how to integrate this measure into clinical practice.

Among SCB, healthy eating, being active, and monitoring had the lowest correct behavior in patients. Similar findings were previously found in Cotonou by Alassani et al. [28] and Wanvoegbe et al. [29] that reported difficulties in compliance with diet (20%), physical activity (55.7%), and glycemic control (7.8%). However, only 9.1% of patients had good therapeutic adherence, contrary to our study, where 51% of participants took diabetes pills, which could be due to our study population following care in private clinics. We also found a direct relationship between DQoL and SCB for healthy eating, problems solving, coping strategies, and risks reduction, suggesting that in addition to nutritional self-care, interventions aimed at improving the diabetic patients’ QoL should be taken into account education as well as personal needs of these patients. These findings confirmed that a patient empowerment, starting with diabetes self-management education, is needed to improve QoL [52,53].

However, our findings should be interpreted cautiously in light of some limitations. A cross-sectional survey design does not allow for the findings’ generalization beyond the sample from which data was gathered. Socioeconomic status or income is a social determinant of disease outcome, but we could not use it in our study due to missing data. Also, data of the two sexes reported were not analyzed separately due to the significant difference in the percentage of female and male participants in the study. Finally, the collected data was based on self-report, and therefore bias may be present due to inaccurate self-reporting or social desirability. Despite these limitations, this study provided the foundation for future studies among persons living with diabetes concerning their diabetes management. This study used standard tools for measuring DQoL and SCB, which allows the measurement of satisfaction, impact, and worrying aspects of life in diabetes. Finally, the study was conducted on T2D patients without considering their diabetic complication history status during the data collection period, which positively or negatively affects their Qol. However, patients who were severely ill were excluded.

## 5. Conclusions

The present study demonstrates a low QoL among 43% of patients with T2D, suggesting that QoL should be included in any modality used for treating diabetic patients in Cotonou. The poor QoL is mainly in the domains of diabetes impact and satisfaction of life. The results also showed that DQoL is influenced by sociodemographic factors such as education, marital status, occupation, family history of diabetes, comorbidities, and social support. In addition, self-care behaviors, including healthy eating, problem-solving, coping strategies, and risk reduction, were identified as significant predictors of the QoL in these patients. Consequently, patient empowerment, starting with diabetes self-management education, is needed to improve DQoL in the surveyed community. However, such programs should target primarily patients with no education, low socioeconomic status, overweight/obesity, and those lacking social support.

## Figures and Tables

**Table 1:** Diabetes-specific quality of life of the study participants.

Items & Domains	Response Categories *n* (%)				
**Satisfaction** Time takes to manage diabetes Time spent getting checkups Time it takes to determine the sugar level Current treatment Knowledge about diabetes Life in general **Mean satisfaction subscore± SD (out of 30 total points)** **% patients > mean**	**Very** **Satisfied**	**Moderately Satisfied**	**Neither Satisfied Nor Dissatisfied**	**Moderately Dissatisfied**	**Very** **Dissatisfied**
	33(11) 25(8.3) 32(10.7) 38(12.7) 15(5.0) 56(18.7)	27(9.00) 29(9.7) 25(8.3) 23(7.7) 68(22.7) 25(8.3)	57(19.0) 77(25.7) 100(33.3) 49(16.3) 55(18.3) 23(7.7)	125(41.7) 137(45.7) 117(39.0) 154(51.3) 100(33.3) 146(48.7)	58(19.3) 32(10.7) 26(8.7) 36(12.0) 62(20.7) 50(16.7)
	**20.6 ± 2.9** **53.00**				
**Impact** Feel pain associated with the treatment Feel physically ill Interfere with the family life Limiting social relationships and friendships **Mean impact subscore (out of 20 total points)** **% patients > mean**	**Never**	**Sometimes**	**Often**	**Frequently**	**Always**
	25(8.33) 28(9.33) 143(47.7) 178(59.3)	28(9.33 21(7.00) 86(28.7) 60(20.0)	50(16.7) 57(19.0) 28(9.3) 21(7.0)	149(49.7) 151(50.3) 14(4.7) 14(4.7)	48(16.00) 43(14.3) 29(6.7) 27(9.0)
	**19.9 ± 1.4** **56.3**				
**Worry** Pass out Body looks differently **Get complications** **Mean worry subscore (out of 15 total points)** **% patients > mean**	**Never**	**Sometimes**	**Often**	**Frequently**	**Always**
	116(38.7) 102(34.0) 63(21.0)	117(39.0) 125(41.7) 142(47.3)	29(9.7) 36(12.0) 43(14.3)	11(3.7) 10(3.3) 16(5.3)	27(9.0) 27(9.0) 36(12.0)
	**6.6 ± 3.3** **32.7**				
**Total quality of life score (out 65 total points)** **% patients > mean**	**38.1 ± 4.1** **43.0**				

**Table 2:** Distribution of diabetes self-care behaviors among study participants.

Items	*n* (%)
**Healthy eating** How many times during the week do you eat away from home? How would you describe your portions of grains and starches? **Mean healthy eating subscore (out of 2 total points)** **% patients > mean**	207(69.0) 108(36.0) **1.33 ± 0.61** **122(40.67)**
**Being active** Do you exercise regularly? How often do you exercise 30 min per week? How sure are you that you can be active? **Mean being active subscore (out of 3 total points)** **% patients > mean**	168(56.0) 106(35.3) **144(48.0)** **1.39 ± 1.20** **139(46.3)**
**Monitoring** Did you test your blood for sugar in the last seven days? Did you test your urine for sugar or ketones in the last seven days? **Mean monitoring subscore (out of 2 total points)** **% patients > mean**	155(51.7) 71(23.7) **0.75 ± 0.71** **149(49.7)**
**Taking medication** Do you take pills for your diabetes? How important is it to you to take your medicines? How sure are you that you can take your medicines? **Mean taking medication subscore (out of 3 total points)** **% patients > mean**	169(56.3) 264(88.0) 237(79.0) **2.27 ± 0.85** **201(67.0)**
**Problems solving** How do you treat a low blood sugar reaction? How do you treat a high blood sugar reaction? **Mean problem solving subscore (out of 2 total points)** **% patients > mean**	58(24.2) 145(48.3) **0.68 ± 0.71** **161(53.7)**
**Coping strategies** How important is being able to problem solve when being faced with every day and/or challenging decisions? Do you feel you can problem solve when faced with every day and/or challenging decisions? **Mean coping strategies subscore (out of 2 total points)** **% patients > mean**	245(81.7) 164(54.7) **1.36 ± 0.73** **153(51.0)**
**Risk reduction** Have you had your blood pressure checked in the last three months? Have you had your cholesterol and triglycerides checked? Have you had an A1c test performed in the last three months? **Mean risk reduction subscore (out of 3 total points)** **% patients > mean**	151(50.33) 127(42.33) 169(56.33) **1.49 ± 1.19** **152(50.7)**
Total diabetes self-care behaviors score (15 total points) % patients > mean	**9.57 ± 2.59** **160(53.3)**

**Table 3:** Socio-demographic factors associated with quality of life.

Variables	Satisfaction		Impact		Worry		Quality of Life	
	OR	95% CI	OR	95% CI	OR	95% CI	OR	95% CI
**Sex** Male Female			1.00 2.41	1.34–5.25				
**Education** No formal education Educated	1.00 0.56	0.29–0.93	1.00 0.26	0.12–0.72	1.00 0.72	0.45–0.98	1.00 0.36	0.19–0.89
**Marital status** Not married Married	1.00 0.39	0.25–0.86	1.00 0.41	0.27–0.93	1.00 0.32	0.18–0.56	1.00 0.52	0.25–0.83
**Occupation** Others Self-employed Government/non-government employee					1.00 1.71 3.87	0.92–3.38 1.58–6.77	1.00 0.54 1.38	0.32–0.97 0.61–4.22
**Family history of diabetes** No Yes	1.00 0.9	0.52–0.97	1.00 0.48	1.06–3.35	1.00 0.61	0.35–0.85	1.00 0.57	0.22–0.88
**Duration of Diabetes (mean ± SD), years** **≤5** **≥6**	1.00 2.60	1.50–4.98	1.00 1.30	1.17–2.29	1.00 1.02	1.01–2.98	1.00 1.15	1.07–2.37
**BMI (mean ± SD), kg/m^2^** Underweight/Normal Overweight/Obese			1.00 1.20	1.32–3.26			1.00 1.34	1.13–2.63
**Comorbidities** No Yes	1.00 2.02	1.32–4.68	1.00 2.26	1.27–4.73	1.00 4.36	1.18–9.76	1.00 1.93	1.22–3.50
**Social support** No Yes	1.00 0.49	0.25–0.86	1.00 0.51	0.36–0.93	1.00 0.63	0.50 = 0.97	1.00 0.50	0.25–0.84
**Diabetes group education class** No Yes	1.00 0.56	0.30–1.68	1.00 0.67	0.43–1.62	1.00 0.62	0.48–1.89	1.00 0.96	0.62–1.70
	R2 = 12.6		R2 = 17.16		R2 = 13.14		R2 = 16.68	

OR = Odds Ratio.

**Table 4:** Self-care behavior factors associated with quality of life of study participants.

Variables	Satisfaction		Impact		Worry		Quality of Life	
Domains	OR	95% CI	OR	95% CI	OR	95% CI	OR	95% CI
**Healthy Eating**	0.79	0.35–0.94	0.55	0.35–0.83	0.60	0.36–0.87	0.45	0.18–0.88
**Being Active**	1.32	0.78–2.67	2.35	1.36–6.12	1.49	0.79–2.96	1.08	0.61–1.96
**Monitoring**	0.55	0.28–1.26	1.73	0.78–2.81	0.96	0.51–1.91	0.60	0.34–1.13
**Taking Medications**	1.75	0.38–1.52	1.40	0.72–2.90	1.86	0.62–2.21	1.22	0.71–1.95
**Problem Solving**	2.23	1.15–4.08	0.32	0.17–0.62	0.68	0.57–0.94	0.45	0.27–0.87
**Coping Strategies**	0.80	0.40–1.06	0.53	0.36–0.75	0.33	0.16–0.62	0.51	0.34–0.94
**Risks Reduction**	0.67	0.45–0.98	0.34	0.34–0.67	0.38	0.19–0.69	0.38	0.26–0.67
	R2 = 18.59		R2 = 22.34		R2 = 20.90		R2 = 20.37	

Controlled with level of education, marital status, occupation, duration of diabetes, family history of diabetes, comorbidities, and social support.

## Data Availability

The datasets used and/or analyzed during the current study are available from the corresponding author on reasonable request.

## Supplementary Materials

The following are available online at https://www.mdpi.com/article/10.3390/ijerph19042360/s1. Table S1: Natividad diabetes self-care behaviors questionnaire; Table S2: Flow of data analysis; Table S3: Evaluation of the revised version of diabetes self-care behaviors; result from confirmatory factor analysis; Table S4: Fit indices for measurement model with 18 items; Table S5: Diabetes self-care behaviors Domains, Indicators, and Aggregate Codes.
