# Supplementary material for: Factors Associated with Quality of Life in Patients with Type 2 Diabetes of South Benin: A Cross-Sectional Study
Source: Int J Environ Res Public Health. 2022 Feb 18;19(4):2360. doi: 10.3390/ijerph19042360 (PMC8871979; doi:10.3390/ijerph19042360)
Supplement: Supplementary file 1 [file ijerph-19-02360-s001.zip › ijerph-1471317-supplementary.pdf]

Table S1. Natividad Diabetes Self Care Behaviors questionnaire

Healthy Eating

1. Height: ________ Weight: ________ What weight are you comfortable at? ________________________
2. Has your weight changed in the past three months? 1 No 2 Yes
   If "yes," I've 1 lost / 2 gained _________ Kg

   Was the weight change intentional? 1 No
   2 Yes____
3. Have you ever received diet counseling? 1 No 2 Yes

   If "yes," describe: ____
4. Do you have a current meal plan? ____If so, what is it?_______________________________________
5. What is your biggest challenge to eating healthily? _____________________________________
6. How many times do you eat per day? 1 Meals _________ 2 Snacks____________
   Times of meals: am__________ noon __________ pm_________ snacks ___________
7. How often do you eat/drink (answer per day or per week):
   Fruit: ______ Juice: ______ Milk: ______ 1 Fat-free 2 1% 3 2% 4 Whole

   Vegetables: ______ Sweets: ______ Sugar-free deserts/drinks ______

   Beverages with sugar: ______ Alcohol: ______ Water: ______ How much a day? _______

   Starches eaten: State number of servings eaten per meal

   1 bread____ 2 potatoes____ 3 beans____ 4 rice____ 5 pasta____ 6 corn/peas____
   7 other_____

   Meats/Proteins: State number of times eaten per week

   1 chicken ____ 2 red meats____ 3 fish____ 4 turkey ____ 5 pork____ 6 eggs____

   7 cheese____ 8 other ___________________

   Cooking Oil/Fat used: 1 Lard/Shortening 2 Butter/Margarine 3 Vegetable/Corn 4 Olive

   5 Peanut
   6 Other____
8. Who does the cooking? ______________ Who usually does the grocery shopping:_________________
9. How many times during the week do you eat away from home? ________________________________
10. How often is your meal away from home:
    1 Cafeteria style: ______ 2 Fast food: ______ 3 Buffet: ______4 Sit-down restaurant: __________

5 Other: ____

11. How is your food usually prepared? 1 Fried 2 Baked 3 Broiled 4 Grilled 5 Steamed
12. How would you describe your portions? 1 Small 2 Average 3 Large
13. How would you describe your appetite? 1 Increased 2 Normal 3 Decreased
14. List any food allergies or intolerance:
____
____

15. Any other special diet needs:
____
____

16. How do mood/stress affect your eating?
____
____

Being Active

1. Do you exercise regularly? 1 No 2 Yes
Type of exercise(s):
____

2. How often do you exercise? _______ How long each time?____________________________________
What time of day do you exercise?
____

List any problems with exercise:
____
____

3. How important is it to you to be active, where 0 is not important at all and 10 is very important?
0 1 2 3 4 5 6 7 8 9 10

4. How sure are you that you can be active, where 0 is not sure and 10 is very sure?
0 1 2 3 4 5 6 7 8 9 10

Monitoring

1. Do you test your blood for sugar? 1 No 2 Yes
If "yes," what blood sugar monitor do you use?
____

Do you have any problems with your monitor? • 1 No 2 Yes
____

How often do you test? 1 Once a day 2 2 or more times a day 3 Once/Twice a week
4 Rarely/Never

Usual results? Mornings:_________ Afternoon:____________ Bedtime:__________

After Meals:____________ Other times:_______________________

2. Do you keep a record? 1 No 2 Yes
3. What is considered a normal blood sugar range?__________________________
4. What are your target numbers?__________________________

5. How often do you have HIGH blood sugar? (250 or more) 1 Daily 2 Several times a week

   3 A few times a month 4 Once in a while 5 Rarely or never 6 Don't know

6. How often do you have LOW blood sugar (70 or less)? 1 Daily 2 Several times a week 3 A few times a month 4 Once in a while 5 Rarely or never 6 Don't know

7. Do you have access to your diabetes supplies? 1 No 2 Yes /Pharmacy _____________________

8. Do you test your urine for sugar or ketones? 1 No 2 Yes /How often?___________________

9. How important is it to you to monitor your blood sugar at least once per day, where 0 is not important at all and 10 is very important?

   0 1 2 3 4 5 6 7 8 9 10

10. How sure are you that you can monitor your blood sugar at least once per day, where 0 is not sure at all and 10 is very sure?

   0 1 2 3 4 5 6 7 8 9 10

Taking Medications

1. Do you take pills for your diabetes? 1 No 2 Yes /What times?__________________________

2. Any side effects from the medications that you know of? 1 No 2 Yes _____________________

3. Do you take any additional nutritional supplements? 1 Vitamins 2 Herbal supplements

   3 Other____

4. Have you ever forgotten to take your diabetes medication? 1 No 2 Yes /How often? _________
5. How important is it to you to take your medicines, where 0 is not important at all and 10 is very important?
   0 1 2 3 4 5 6 7 8 9 10

6. How sure are you that you can take your medicines, where 0 is not sure at all and 10 is very sure?
   0 1 2 3 4 5 6 7 8 9 10

Problem Solving

1. Have you ever had a low blood sugar reaction with symptoms such as sweating, weakness, anxiety, trembling, hunger or headache? 1 No 2 Yes

   If "yes," How many days in the last month?

____

   How did you treat it?

____

   Did you require assistance or hospitalization for it? 1 No 2 Yes

____

7. Does your family or friends know that you are diabetic? 1 No 2 Yes 3 Don't know
8. Have you ever had high blood sugar with symptoms such as thirst, dry mouth and skin, increased sugar in the urine, less appetite, nausea, or fatigue? 1 No 2 Yes 3 Don't know
   If "yes, How many days in the last month?

____

   What did you do to treat it?

____

   Have you ever been hospitalized for very high blood sugars? 1 No 2 Yes:
When/Where:___________

____

9. When you are sick or cannot eat usual food, how do you take care of yourself?
   1 Replace usual food with carbohydrate or sugar 2 Take diabetes medication

   3 Check ketone levels •4 Check blood sugar more often 5 Drink more water

   6 Contact healthcare provider 7 Do nothing
   8 Other______________________________

Stress

1. Is there much stress in your life? 1 No 2 Yes

   If "yes," explain: ____

2. What do you do to handle stress in your life? ____

3. How important is being able to problem solve when being faced with everyday and/or challenging decisions, where 0 is not important at all and 10 is very important?

   0 1 2 3 4 5 6 7 8 9 10

4. Do you feel you can problem solve when faced with everyday and/or challenging decisions, where 0 is not sure at all and 10 very sure?

   0 1 2 3 4 5 6 7 8 9 10

   5. Do you perceive problems with your diabetes management, where 0 is none perceived and 10 is perceive many?

   0 1 2 3 4 5 6 7 8 9 10

Healthy Coping

1. How would you describe your general health? 1 Good 2 Fair 3 Poor
2. Is your health important to you? 1 All the time 2 Sometimes 3 Only when ill 4 Not at all
3. How do you feel about having diabetes? ____
4. Do you feel diabetes is serious? 1 No 2 Yes
5. Do you feel you can control your diabetes? 1 Yes 2 No
6. Is good control worth it? 1 Yes 2 No
7. My diabetes has caused problems in the following areas:

1 Family life/social activities 2 Work/school 3 Sports/exercise 4 Sexual relations 5 Finances
6 Contentment 7 Travel 8 Other: ______________________________

8. Are you currently experiencing any of the following? 1 No problems

   1 Recent death 2 Separation 3 Divorce 4 Illness 5 Unemployment 6 Financial difficulties

   7 Housing problems 8 Depression symptoms 9 Loneliness 10 Confusion

   11 Thoughts of hurting yourself 12 Other: ____

9. Do you have history of depression? 1 No 2 Yes /How often do you feel depressed?

   1 A lot 1 Some 1 A little 1 Not at all

Reducing Risks

1. How often do you have your eyes checked by an eye doctor? ___________________________
   Date of last exam (with drops in the eyes) : ____

2. Do you wear glasses? 1 No 2 Yes/For what? ___________________________________
3. Have you noticed any changes in your skin recently? 1 No 2 Yes
   If "yes," please describe: ____

4. How often do you check your feet at home? 1 Daily 2 Weekly 3 Never 4 Other ______________
   Date of last foot exam by doctor: ____

5. How often do you have a dental checkup? ____________ Date of last checkup: ___________________
6. Have you had your blood pressure checked? 1 No 2 Yes When:___________________
7. Have you had a fasting glucose (blood sugar) checked? 1 No 2 Yes When:________________
8. Have you had your cholesterol and triglycerides checked? 1 No 2 Yes When: ______________
9. Have you had an A1c test done? 1 No 2 Yes When: __________________
10. How sure are you that you can get the help you need to prevent or reduce problems related to diabetes, where 0 is not sure at all and 10 is very sure?
   0 1 2 3 4 5 6 7 8 9 10

Table S2: Flow of data analysis

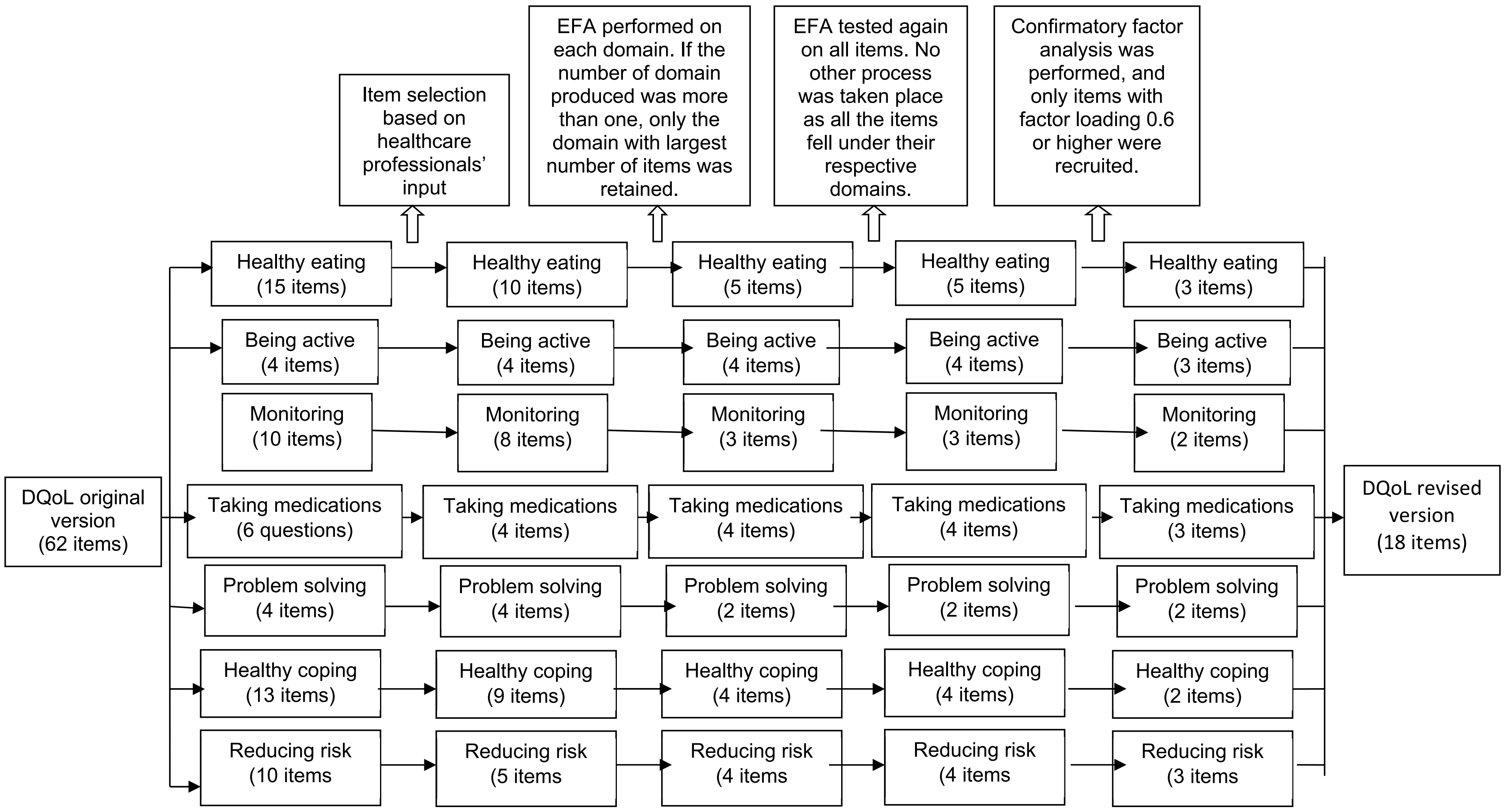

Table S3. Evaluation of the revised version of diabetes self-care behaviors; result from confirmatory factor analysis

**Items**	**Factor loadings**
**Healthy eating**	
1. How often is your meal away from home?	0.747
2. How would you describe your grain and starches portions?	0.792
**Being active**	
1. Do you exercise regularly?	0.835
2. How often do you exercise 30 minutes a week?	0.829
3. How important is it to you to be active?	0.765
**Monitoring**	
1. Did you test your blood for sugar in the last seven days?	0.842
2. Did you test your urine for sugar or ketones in the last seven days?	0.744
**Taking medications**	
1. Did you take pills for your diabetes?	0.683
2. How important is it to you to take your medicines?	0.829
3. How sure are you that you can take your medicines?	0.786
**Problem solving**	
1. How did you treat a low blood sugar reaction?	0.793
2. What did you do to treat high blood sugar?	0.706
**Healthy coping**	
1. How important is being able to problem solve when being faced with everyday and/or challenging decisions?	0.818
2. Do you feel you can problem solve when faced with everyday and/or challenging decisions?	0.747
**Reducing risk**	
1. Did you have your blood pressure checked in the last three months?	0.757
2. Did you have your cholesterol and triglycerides checked in the last three months?	0.846
3. Did you have your hemoglobin A1c test done in the last three months?	0.851

Table S4. Fit indices for measurement model with 18 items

Fit indices	Cutt-off value	18 items
CF fit	p>0.05	0.832
RMSEA	<0.05	0.038
SRMR	≤0.08	0.050
CFI	≥0.95	0.98
TLI	≥0.95	0.95

Table S5. Diabetes self-care behaviors Domains, Indicators, and Aggregate Codes

**Domains & Questions**	**Responses categories**	**Aggregate code used**
**Healthy eating**		
3. How often is your meal away from home?	1•Never 2•1- 3 times a week 3• More than 3 times a week	Code=1 if Q=1 or 3 Code=0 if Q=2
4. How would you describe your portions of grains and starches?	1•Less than 1 cup 2•1 cup 3•Larger than 1 cup	Code=1 if Q=1 or 2 Code=0 if Q= 3
**Being active**		
4. Do you exercise regularly?	1•No 2•Yes	Code=1 if Q=2 Code=0 if Q=1
5. How often do you exercise 30 minutes a week?	1• Less than 3 times a week 2• 3 and more times a week	Code=1 if Q=2 Code=0 if Q=1
6. How important is it to you to be active, where 0 is not important at all and 10 is very important?	0-10	Code=1 if Q=7-10 Code=0 if Q=0-6
**Monitoring**		
3. Do you test your blood for sugar in the last seven days?	1•No 2•Yes	Code=1 if Q=2 Code=0 if Q=1
4. Do you test your urine for sugar or ketones in the last seven days?	1•No 2•Yes	Code=1 if Q=2 Code=0 if Q=1
**Taking medications**		
4. Do you take pills for your diabetes?	1•No 2•Yes	Code=1 if Q=2 Code=0 if Q=1
5. How important is it to you to take your medicines, where 0 is not important at all and 10 is very important?	0-10	Code=1 if Q=7-10 Code=0 if Q=0-6
6. How sure are you that you can take your medicines, where 0 is not sure at all and 10 is very sure?	0-10	Code=1 if Q=7-10 Code=0 if Q=0-6
**Problem solving**		
3. How did you treat a low blood sugar reaction?	1 Eat or drink 15 to 20 grams of fast-acting carbs. 2 Others	Code=1 if Q=1 Code=0 if Q=2
4. What did you do to treat high blood sugar?	1 Get physical 2 Take your medication as directed. 3 Follow your diabetes-eating plan. 4 Check your blood sugar 5 Do nothing 6 Other	Code=1 if Q=1-4 Code=0 if Q=5 or 6
**Healthy coping**		

3. How important is being able to problem solve when being faced with everyday and/or challenging decisions, where 0 is not important at all and 10 is very important?<br>4. Do you feel you can problem solve when faced with everyday and/or challenging decisions, where 0 is not sure at all and 10 very sure?	0-10<br><br><br><br>0-10	Code=1 if Q=7-10<br>Code=0 if Q=0-6<br><br><br>Code=1 if Q=7-10<br>Code=0 if Q=0-6
**Reducing risk**<br>4. Did you have your blood pressure checked in the last three months?<br>5. Did you have your cholesterol and triglycerides checked in the last three months?<br>6. Did you have an A1c test done in the last three months?	1•No 2•Yes	Code=1 if Q=2<br>Code=0 if Q=1
